# Modelling maternal obesity: the effects of a chronic high-fat, high-cholesterol diet on uterine expression of contractile-associated proteins and *ex vivo* contractile activity during labour in the rat

**DOI:** 10.1042/CS20150539

**Published:** 2015-12-17

**Authors:** Ronan Muir, Jean Ballan, Bethan Clifford, Sarah McMullen, Raheela Khan, Anatoly Shmygol, Siobhan Quenby, Matthew Elmes

**Affiliations:** * Division of Nutritional Science, School of Bioscience, University of Nottingham, Sutton Bonington Campus, Loughborough LE12 5RD, England, U.K.; † Graduate School of Medicine, University of Nottingham, Royal Derby Hospital, Uttoxeter Road, Derby DE22 3DT, England, U.K.; ‡ Reproductive Health, University of Warwick, Coventry, Warwickshire CV2 2DX, England, U.K.; § Biomedical Research Unit in Reproductive Health, University Hospital Coventry and Warwickshire, Coventry, Warwickshire CV2 2DX, U.K.

**Keywords:** connexin 43, labour, maternal obesity, oxytocin, oxytocin receptor, uterine contractility.

## Abstract

Maternal obesity is associated with prolonged and dysfunctional labour and emergency caesarean section, but the mechanisms are unknown. The present study investigated the effects of an adiposity-inducing high-fat, high-cholesterol (HFHC) diet on uterine contractile-associated protein (CAP) expression and *ex vivo* uterine contractility in term non-labouring (TNL) and term labouring (TL) rats. Female rats were fed either control chow (CON *n*=20) or HFHC (*n*=20) diet 6 weeks before conception and during pregnancy. On gestational day 21 (TNL) or day 22 (TL) CON and HFHC (*n*=10) rats were killed to determine plasma cholesterol, triacylglycerol and progesterone concentrations and collection of myometrium for contractility studies and expression of CAPs caveolin-1 (Cav-1), connexin-43 (CX-43) and it's phosphorylated form (pCX-43), oxytocin receptor (OXTR) and cyclooxygenase-2 (COX-2). HFHC feeding increased visceral fat (*P*≤0.001), plasma cholesterol (*P*≤0.001) and triacylglycerol (*P*=0.039) concentrations. Stage of labour effected uterine expression of CAV-1 (*P*<0.02), pCX43 and COX-2 (both *P*<0.03). CAV-1 and pCX43 decreased but COX-2 increased with parturition. Significant diet- and labour-stage interactions were evident for CX-43 and pCX43 (*P*<0.03 and *P*<0.004 respectively). CX-43 decreased with TL in HFHC animals but was unaltered in CON. pCX-43 fell with labour in CON but remained high in HFHC. OXTR expression was significantly higher in HFHC compared with CON animals (*P*<0.03). Progesterone was higher in HFHC rats at term (*P*<0.014) but fell significantly with labour to similar concentrations as CON. Contractility studies identified synchronous contractions of stable amplitude in lean animals, but unstable asynchronous contractions with obesity. Uterine dose response to oxytocin was blunted during labour in HFHC rats with a log EC_50_ of −8.84 compared with −10.25 M in CON for integral activity (*P*<0.05). In conclusion, our adiposity model exhibits adverse effects on contractile activity during labour that can be investigated further to unravel the mechanisms causing uterine dystocia in obese women.

## Abstract (precis)

Modelling maternal obesity in rats adversely affected steroid synthesis, uterine contractile associated protein expression and *ex-vivo* uterine contractility during labour. This maternal obesity model can be utilized further to unravel the mechanisms causing uterine dystocia in obese women.

## CLINICAL PERSPECTIVE

• Maternal obesity is associated with a prolonged and dysfunctional labour and a significant increase in the risk of caesarean delivery but the mechanism is currently unknown.
• The current study sets up a translational animal model of maternal obesity to begin to unravel the mechanisms involved and finds that increased abdominal adiposity led to asynchronous contractions and adverse alterations in uterine contractile protein expression and progesterone production in comparison with lean animals.
• This obesity model can help identify the mechanism(s) responsible for uterine dystocia in obese women. Dietary intervention or drug therapy may also be identified to help sustain or augment labour in this high-risk pregnancy group and decrease the risk of morbidity and mortality to the mother and child, as well as decreasing NHS costs.

## INTRODUCTION

A previous study has identified that over 20% of the female population in the U.K. are obese and this number is expected to double by 2050 [1]. This rapid rise in obesity is already placing a huge strain on maternity services nationwide [2] as obese patients are at greater risk of complications including, pre-eclampsia, post-partum haemorrhage [3–6] and prolonged and dysfunctional labour with increased risk of emergency caesarean delivery [3,4]. Caesarean delivery rates in nulliparous singleton pregnancies between obese and normal weight women are 29% and 15% respectively [5]. The increasing number of caesarean sections due to maternal obesity is also placing a huge financial strain on the National Health Service (NHS). A single caesarean delivery will cost £1530 more than a single spontaneous vaginal delivery [2] and if caesarean delivery numbers could be reduced annually by 1%, it would save over £5 million [2]. This excludes the increased cost of longer hospital stay, recovery and post-operative infection [6]. The biological mechanism underpinning uterine dystocia associated with maternal obesity is yet to be determined. Earlier research has illustrated that feeding a high-fat, high-cholesterol (HFHC) diet prior to and during pregnancy in rats has adverse effects on uterine expression of key contractile-associated proteins (CAPs) and pregnancy-related hormones during labour that are essential to the timing and control of parturition [7]. This study focused on the contractile proteins caveolin-1 (CAV-1), connexin 43 (CX-43) and cyclooxygenase-2 (COX-2) during labour. CAV-1 is the structural component of caveolae [8], ω-shaped cholesterol-rich invaginations of cell membranes that act as platforms for the coding of intracellular signals [9] and transduction pathways that regulate contractile activity. Evidence to support this is that cav-1 knockout mice exhibit impaired smooth muscle relaxation [10]. CX-43 is the major myometrial gap junction protein that facilitates intracellular propagation of electrical impulses [11] and synchronizes myometrial contractions, whereas COX-2 is responsible for the synthesis of prostaglandins PGF_2α_ (prostaglandin PGF_2α_) and prostaglandin PGE_2_ (PGE_2_) [12] which stimulate [13] and relax [14] myometrial contractions respectively. These findings suggest that HFHC-induced increases in adiposity could have a negative impact upon uterine contractility. The present study will expand further on these findings by investigating the effects of a HFHC diet on uterine expression of CAP during both term non-labouring (TNL) and term labouring (TL), but also the effect the HFHC diet has on spontaneous and stimulated myometrial contractile activity *ex vivo*. This approach tested the hypothesis that a HFHC diet would negatively affect uterine contractility and uterine CAP expression within TNL and TL animals.

## MATERIALS AND METHODS

### Animals and experimental design

All animal work was approved by local research ethics committee and completed within the animal research facilities at the University of Nottingham and carried out under the Animals Scientific Procedures Act (ASPA) 1986. Forty weanling virgin female Wistar rats (*Rattus rattus*) weighing approximately 60 g (Charles River) were pair housed under normal conditions (12-h light/dark photoperiod, 21±5°C room temperature, 55±5% relative humidity, food and water access *ad libitum*) and randomly assigned to be fed either a standard laboratory chow (Harlan Laboratories, *n*=20) or HFHC diet (*n*=20) as previously published [7]. Each rat was maintained on their respective diets for 6 weeks prior to mating with stud Wistar males (Charles River). Pregnancy was confirmed upon discovery of a semen plug and recorded as gestational day 0. Following successful mating, pregnant females were maintained on their respective diet throughout gestation. Animals in each dietary group were killed by CO_2_ asphyxiation and cervical dislocation at two time points; TNL at gestational day 21 (*n*=10) or day 22 TL (*n*=10), following delivery of the first pup to collect plasma and uterine tissue. Blood was collected via cardiac puncture into EDTA-coated tubes (Sarstedt). Plasma separated via centrifugation at 13000 ***g*** for 10 min and snap frozen in liquid nitrogen. The uterus was dissected and split into two horns. The right horn was washed and stored in ice-cold Krebs–Henseleit buffer (NaCl 118 mM, KCl 4.7 mM, KH_2_PO_4_ 1.2 mM, MgSO_4_·7H_2_O 1.2 mM, NaHCO_3_ 25 mM, CaCl_2_·2H_2_O 1.25 mM and glucose 11 mM that had been gassed with 95% O_2_/5%CO_2_) and used for *ex vivo* contractility studies. To standardize sample collection, uterine strips were taken from the same region of the right uterine horn in all animals. The left uterine horn was snap frozen in liquid nitrogen and stored at–80°C. It is important to note that some animals did not conceive and were not pregnant at the time of killing; in addition, some myometrial strips did not stabilize or produce spontaneous contractions, so sample sizes for each experiment are stated clearly within each figure legend.

### *Ex vivo* contractility study

Small strips (∼10×5 mm) of circular myometrium were dissected from the uterine horn from each animal and mounted in a separate 25 ml of organ bath (Letica, AD Instruments) filled with physiological saline solution (PSS; 119 mM NaCl, 4.7 mM KCl and 2.4 mM MgSO_4_, 25 mM NaHCO_3_ and 1.18 mM KH_2_PO_4_, 5.5 mM glucose and 1.6 mM CaCl_2_) warmed to 37°C and gassed with 95% O_2_ and 5% CO_2_. Each strip was set to a resting tension of 20 mN and contractile activity of each strip was recorded using isometric force transducers connected to a bridge amplifier which in turn was connected to a dedicated data acquisition system (Powerlab/8SP, AD Instruments) and recorded and analysed by Lab Chart Software (version 6; Powerlab ASD Instruments). Myometrial strips were then left to equilibrate for 30-40 min. Following equilibration, 30 min spontaneous baseline contractile function was determined before the accumulative addition of oxytocin (OXT; 10^−12^–10^−5^ mol/l) or PGF_2α_ (10^−10^–10^−3^ mol/l) applied at 10-min intervals. The resultant contractile activity measured included activity integrals (area under the time–force curve), mean amplitude and frequency of contractions. Viability of myometrial strips was checked at the end of each experiment by recording contractile responses to administration of 0.1 M KCl.

### Total cholesterol and triacylglycerol assays

Total cholesterol and triacylglycerols in the maternal plasma were assayed through a commercial kit (Thermoscientific fisher) according to the manufacturers' instructions.

### Plasma progesterone assay

Maternal plasma progesterone concentrations were determined using an ELISA (Ridgeway Science) according to the manufacturer's instructions.

### Western blotting

For analysis of uterine expression of Cav-1, CX-43, phosphorylated CX-43 (pCX43), oxytocin receptor (OXTR) and COX-2, uterine tissue was ground to a powder in liquid nitrogen, with 300 mg homogenized briefly for 30 s in ice-cold extraction buffer [32.5 mM Tris/HCl, 3.08 mM EDTA, pH 7.5, and protease inhibitor cocktail III (Calbiochem)]. Homogenates were then split into two parts for analysis of each protein. Homogenate for COX-2 underwent centrifugation at 13000 ***g*** whereas Cav-1, CX-43, pCX43 and OXTR were spun at 3500 ***g*** for 15 min at 4°C to isolate the supernatant. The total protein concentration of the supernatant was determined using the Bio-Rad total protein assay reagent (Bio-Rad) according to the manufacturer's instructions. Sample concentrations were standardized (2, 20 and 22 μg/μl) using SDS mixture (62.5 mM Tris, pH 6.8, 2% SDS, 10% glycerol, 0.02% Bromophenol Blue, 150 mM DTT), boiled for 5 min (samples were not boiled for the detection of COX-2), before equal protein quantities of each sample were separated by SDS PAGE (Cav-1 2 μg/μl, CX-43 and OXTR 22 μg/μl, COX-2 20 μg/μl and pCX43 16 μg/μl). Proteins were transferred to nitrocellulose membrane (Hybond-C extra, Amersham Bioscience) and probed overnight at 4°C with primary antibodies to CAV-1 (1:1000) and CX-43 (1:2500; Cell Signalling Technology), OXTR (1:10000; Santa Cruz Biotechnology), COX-2 (1:3000) and pCX43 (1:2000; Abcam). Membranes were then incubated with a horseradish peroxidise secondary antibody conjugated to rabbit or mouse IgG diluted to a working concentration (GE Healthcare) with 2% ECL prime blocking agent for 1 h at room temperature (secondary antibody dilutions for CAV-1, CX-43, pCX43, OXTR and COX-2 were 1:400, 1:25000, 1:8000, 1:80000, 1:10000 and 1:40000 respectively). Bands were developed on high performance chemiluminescence film (Hyperfilm ECL) using ECL reagent (GE Healthcare). Equal protein loading was verified by reprobing blots with β-actin (Sigma–Aldrich; primary antibody dilution 1:1000 and secondary antibody 1:40000). Where the contractile protein of interest was the same molecular weight as β-actin, membranes were stripped for 30 min with Restore PLUS Western Blot Stripping Buffer (ThermoFisher Scientific) before reprobing. Densitometric analysis of band intensity was performed using a Biorad Gel Doc XR imaging system and Quantity One 1D analysis software.

### Statistics

All data were analysed using the Statistical Package for Social Science (Version 21; SPSS Inc) and expressed as the mean value with standard error, where *P*≤0.05 was considered statistically significant. All data were checked for homogeneity and, if not homogenous, data were transformed to achieve a normal distribution. The effect of a HFHC diet on study measurements unless specified was determined by two-way ANOVA. Statistical analysis to determine the effect of a HFHC diet on plasma total cholesterol, triacylglycerol and progesterone concentrations was achieved using a Kruskal–Wallis test with post hoc Mann–Whitney U test and Bonferonni analysis, as equal variance could not be achieved. Dose response curves to OXT and PGF_2α_ were fitted using the standard least squares (ordinary) fit method. The effects of maternal diet on logEC_50_ values and steepness of the curve (Hill slope) for integral activity were analysed by GraphPad Prism (Version 6; GraphPad Inc) using a sigmoidal dose–response (variable slope) curve to test the null hypothesis that logEC_50_ and Hill slope for the same for each data set. All graphs were produced using Graph Pad Prism (Graph Pad Version 6).

## RESULTS

### Maternal weight gain and litter size

Compared with control chow (CON) animals, those fed the obesity-inducing HFHC diet had a significantly greater body weight at term (Table 1). The increased body weight in HFHC animals was associated with peri-renal and gonadal fat depots being 2-fold higher than CON rats. (Peri-renal, CON 6.68 g ± 0.83 compared with HFHC 13.19 g ± 1.35, *P*≤0.001; gonadal, CON 6.07 g ± 0.64 compared with HFHC 11.00 g ± 1.73, *P*<0.032). Litter size was not affected by maternal diet (Table 1).

**Table 1:** Summary effects of a CON (*n*=17) or HFHC (*n*=15) diet on physiological parameters during pregnancy

Category	Control diet (*n*=17) (Mean ± S.E.M.)	HFHC diet (*n*=15) (Mean ± S.E.M.)	*P-*value
Final weight (g)	416.87±5.77	456.37±12.69	0.012
Peri-renal fat mass (g)	6.68±0.83	13.19±1.35	≤0.001
Gonadal fat mass (g)	6.07±0.64	11.00±1.73	<0.032
Litter size (*n*)	14.06±0.91	13.60±0.82	0.597
Plasma total cholesterol (mmol/l)	2.63±0.08	9.00±0.44	≤0.001
Plasma triacylglycerol (mmol/l)	1.24±0.17	1.67±0.18	0.039

### Plasma lipid profiles

Determination of total cholesterol and triacylglycerol concentration in the maternal plasma of CON- and HFHC-fed animals identified that feeding of the HFHC diet significantly increased circulating concentrations of total cholesterol (*P*≤0.001) and triacylglycerols (*P*=0.039; Table 1). Furthermore, splitting data by gestational age revealed that HFHC-fed animals had significantly higher plasma concentrations of cholesterol at TNL and TL when compared with CON animals (TNL CON 2.5±0.1 mmol/l compared with HFHC 9.8±0.4 mmol/l; TL CON 2.7± 0.1 mmol/l compared with HFHC 8.5±0.6 mmol/l, both *P*≤0.001). A comparison of plasma triacylglycerols at TL revealed that HFHC feeding significantly raised plasma triacylglycerol levels relative to CON rats (*P*<0.001, CON 0.56±0.06 compared with HFHC 1.3±0.2 mmol/l). Interestingly, there was a decrease in plasma triacylglycerol concentrations from TNL to TL across both dietary groups, with TNL concentrations observed to be 1.7±0.2 mmol/l and 2.3±0.06 mmol/l in CON- and HFHC-fed rats respectively.

### Plasma progesterone concentrations

**Figure 1:**
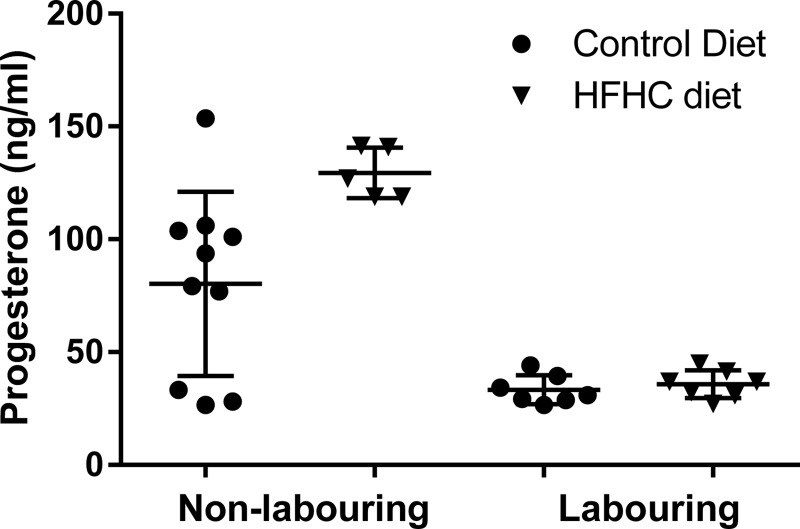
The effect of a CON or HFHC diet on maternal plasma progesterone concentrations at TNL and during TL Sample sizes CON (TNL=10, TL=7) and HFHC (TNL=5, TL=7). Values are means ± S.E.M.

The progesterone ELISA identified that maternal plasma progesterone concentrations of CON- and HFHC-fed animals substantially decreased with the onset of labour (Figure 1); this decrease was significant in HFHC-fed animals (*P*=0.003) but did not quite reach significance in CON rats (*P*=0.051). A comparison between dietary groups at term identified that the HFHC-fed rats had significantly higher concentrations of progesterone than CON (*P*<0.014; 129.44±5.00 compared with 80.26±12.92 ng/ml; *P*<0.014); however, upon onset of labour progesterone concentrations were similar in both dietary groups (CON 33.33±2.41 compared with HFHC 35.75±2.33 ng/ml).

### Uterine expression of contractile-associated proteins

**Figure 2:**
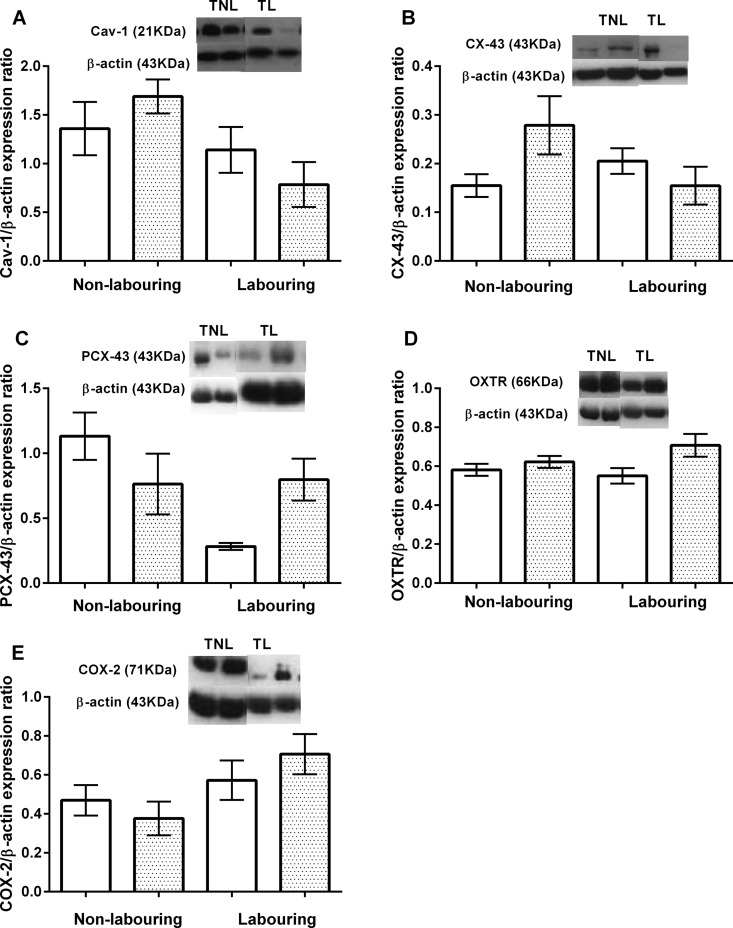
The effect of a CON or HFHC diet upon CAP expression in the TNL or TL uterus (**A**) Cav-1, (**B**) CX-43, (**C**) pCX43, (**D**) OXTR and (**E**) COX-2. Sample sizes CON (TNL=9, TL=8) and HFHC (TNL=6, TL=8). Values are means ± S.E.M. White and shaded bars represent CON and HFHC rats respectively.

Western blot analysis of Cav-1 expression within the uterus identified a significant effect of stage of labour (*P*<0.015), where uterine Cav-1 expression decreased between TNL and TL animals (Figure 2A). Uterine expression of CX-43 was unaffected by diet or stage of labour but there was a significant interaction between the two (*P*<0.02). The HFHC diet decreased uterine expression of CX-43 between TNL and TL, whereas animals sustained on the control diet displayed no change in CX-43 expression with labour onset (Figure 2B). Expression of pCX43 within the uterus was significantly affected by stage of labour (*P*<0.03) but also exhibited a significant interaction with diet (*P*<0.004). In lean CON rats, pCX43 decreased with term labour but remained high during labour in the obese HFHC-fed rats (Figure 2C). Similarly, uterine COX-2 expression was significantly altered by stage of labour (*P*<0.031), increasing in expression during TL (Figure 2E). In contrast, there was an effect of diet on uterine expression of OXTR which was significantly higher in the HFHC fed animals compared with CON (*P*=0.027; Figure 2D).

### Effects of a HFHC diet on spontaneous uterine contractile activity

**Figure 3:**
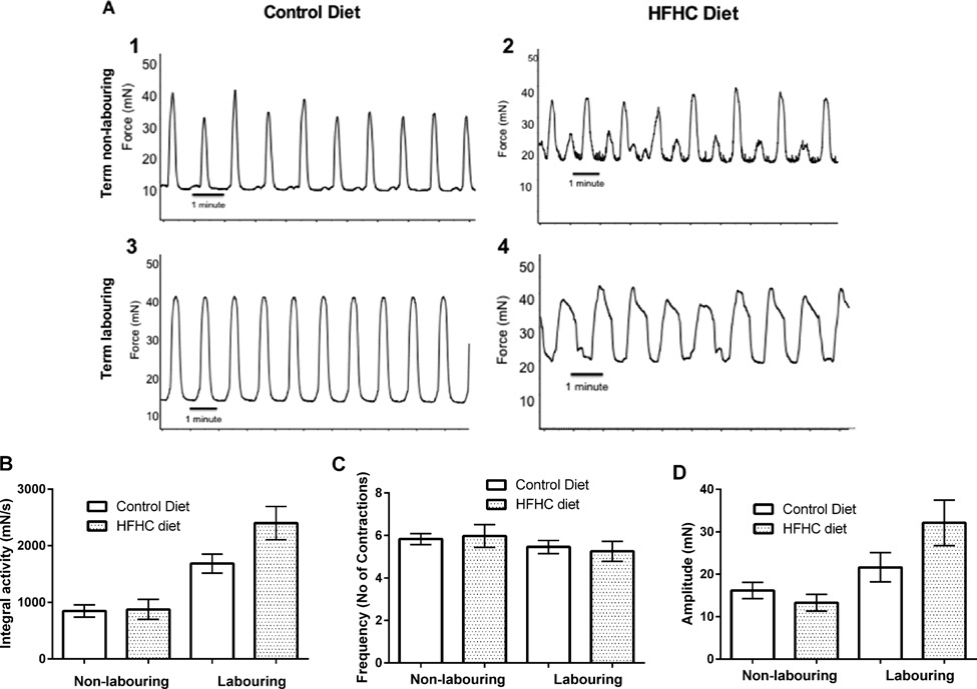
Contractile activity measurements from uterine strips of rats fed either a CON or HFHC diet obtained during TNL and TL (**A1–A4**) Representative traces of spontaneous contractile activity *ex vivo*, (**B**) 30 min integral activity, (**C**) mean frequency of contractions during 30 min baseline and (**D**) mean amplitude of uterine contractions during 30 min baseline recording. Sample sizes CON (TNL=9, TL=8) and HFHC (TNL=6, TL=8). Values are means ± S.E.M.

Qualitative analysis of spontaneous uterine contractile activity in CON animals identified that TNL tissue displayed synchronous plateau contractions of short duration that fluctuated in amplitude (Figure 3A1). Uterine strips obtained from CON rats during TL exhibited the same synchronous plateau contractions but the amplitude was stronger and more consistent during baseline recording (Figure 3A3). In contrast, uterine tissue obtained from HFHC-fed rats during TNL produced asynchronous spike bundle contractions with sizeable fluctuations in amplitude (Figure 3A2). The HFHC fed animals displayed increased duration of individual contractions and decreased amplitude variability during labour. Although there seemed to be an improvement in the contraction phenotype with the onset of labour, the asynchronous contraction phenotype persisted (Figure 3A4). This data suggest that increased maternal adiposity and or high fat feeding seem to have a detrimental impact upon the contraction phenotype at term and during labour. Statistical analysis identified that integral uterine activity was not effected by diet, but was significantly altered with stage of labour, integral activity actually doubling from TNL to TL (*P*<0.001; Figure 3B). Maternal diet and stage of pregnancy had no significant effect on the frequency of spontaneous contractions (Figure 3C). Interestingly, the amplitude of uterine contractions was not affected by diet but did increase significantly from TNL to TL in both CON- and HFHC-fed rats (*P=*0.009; Figure 3D).

### Effects of HFHC diet on uterine contractile response to OXT and PGF_2α_ *ex vivo*

**Figure 4:**
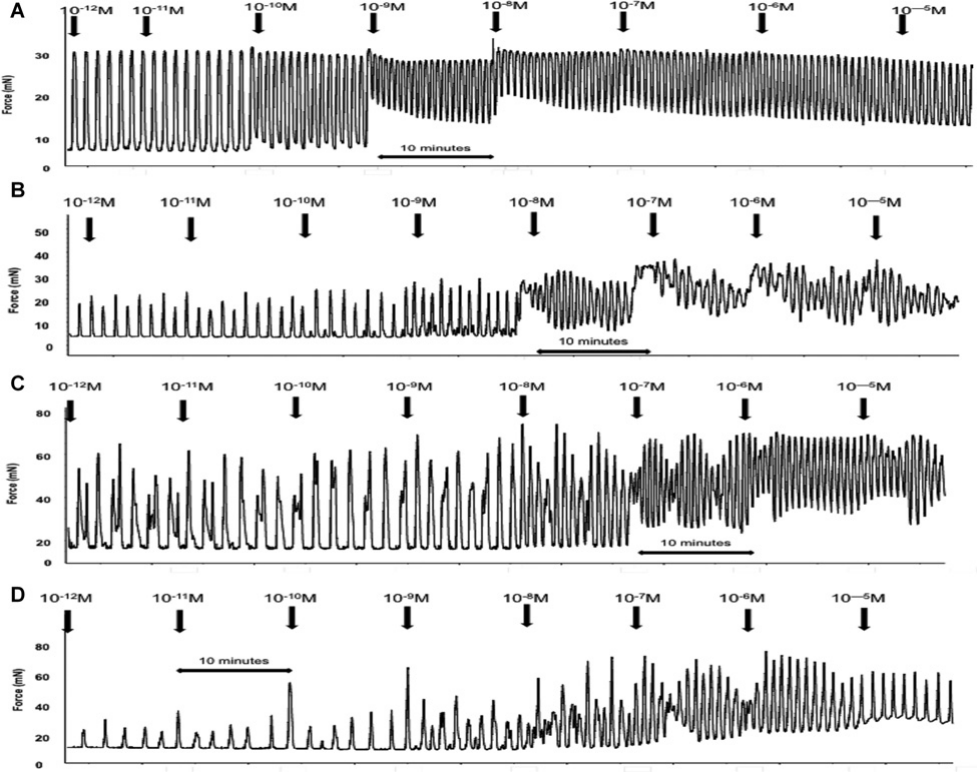
Representative *ex vivo* uterine contractility traces from accumulative increases in oxytocin concentration from (**A**) a CON TL rat, (**B**) a CON TNL rat, (**C**) a HFHC TL rat and (**D**) a HFHC TNL rat

**Figure 5:**
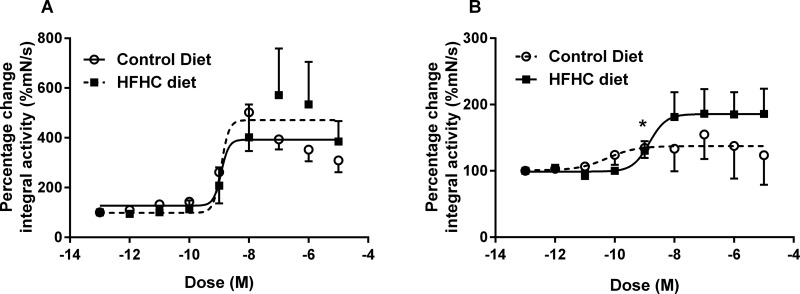
Uterine contractile response *ex vivo* to increasing doses of oxytocin from CON or HFHC fed rats during TNL (**A**) or TL (**B**) Sample sizes CON (TNL=7, TL=7) and HFHC (TNL=5, TL=6). Values are means ± S.E.M. *Signifies significant difference between CON and HFHC rats at the *P*<0.05 level.

**Figure 6:**
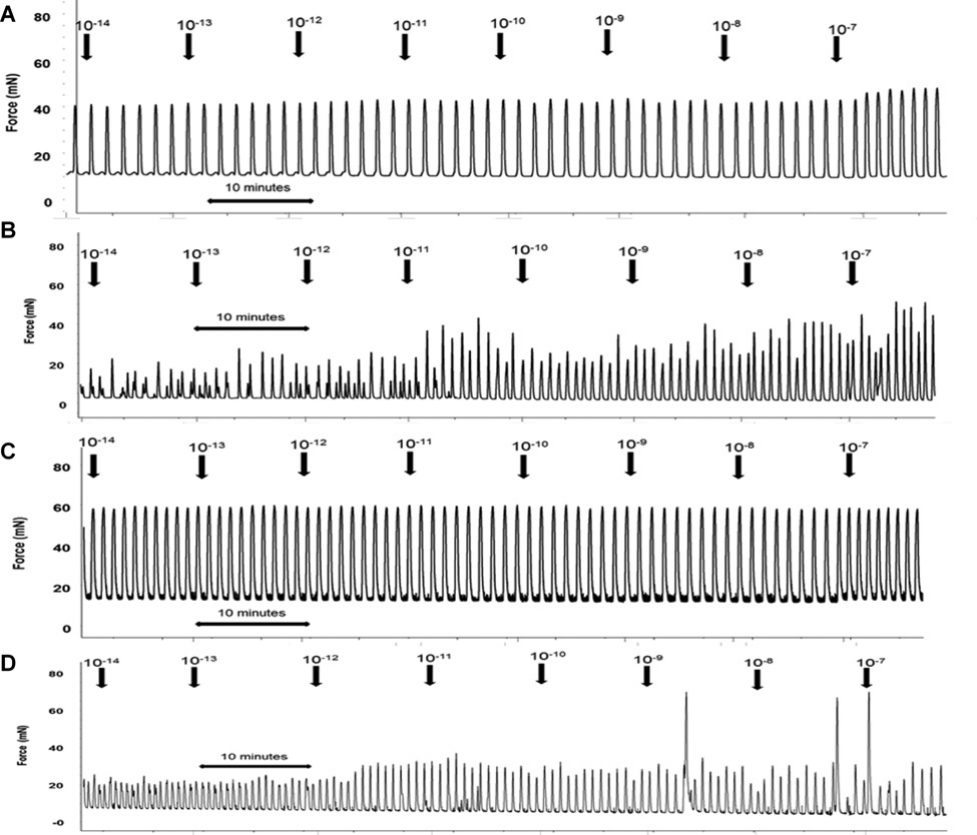
Representative *ex vivo* uterine contractility traces from accumulative increases in PGF_2α_ concentration from (**A**) a CON TL rat, (**B**) a CON TNL rat, (**C**) a HFHC TL rat and (**D**) a HFHC TNL rat

**Figure 7:**
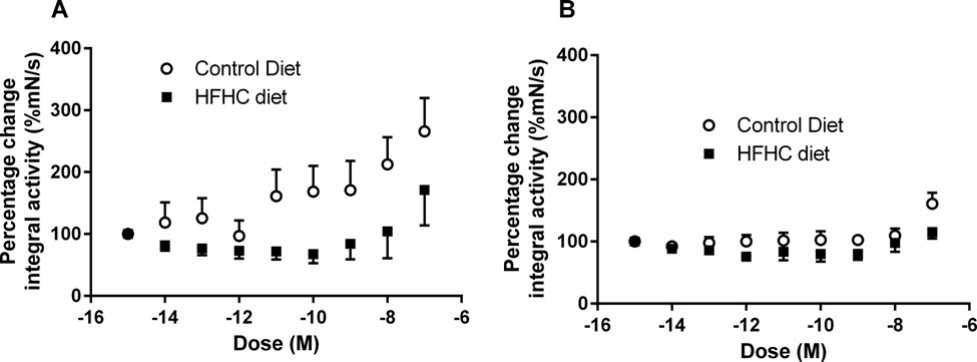
Uterine contractile response *ex vivo* to increasing doses of PGF_2α_ from CON- or HFHC-fed rats during TNL (**A**) or TL (**B**) Sample sizes CON (TNL=4, TL=5) and HFHC (TNL=5, TL=7). Values are means ± S.E.M.

With evidence suggesting that HFHC-induced adiposity alters spontaneous uterine activity, it was important to determine the myometrial response to OXT and PGF_2α_. Application of increasing concentrations of OXT resulted in constriction and increased basal tone of myometrial strips that was associated with decreased amplitude and increased frequency of phasic contractions; a response that occurred in both CON- and HFHC-fed animals during both TNL and TL. Interestingly, the increase in basal tone in response to OXT in HFHC appeared to occur at concentrations of 10^−10^ M in CON- but at 10^−9^M in HFHC-fed rats (Figures 4A–4D), providing some evidence that myometrium from HFHC-fed rats may require a higher concentration of OXT to elicit the same contractile response. It was evident that increasing the concentration of oxytocin also improved synchronization of contractions of uterine strips from HFHC-fed rats during both TNL and TL (Figures 4C and 4D). Spontaneous contractile activity of uterine strips from both CON- and HFHC-fed rats responded to increasing doses of OXT with increases in integral activity. The response to increasing doses of oxytocin was similar between CON and HFHC animals at term; however, HFHC rats exhibited a blunted response requiring higher concentrations of OXT than CON to increase integral activity during labour (Figures 5A and 5B). Sigmoidal dose–response analysis confirmed that logEC_50_ and Hill slope were significantly different between CON and HFHC animals during labour. CON rats were more sensitive to OXT with a log EC_50_ of–10.25 M compared with–8.84 M in HFHC (*P*<0.05). In contrast, HFHC animals had a significantly steeper Hill slope (*P*<0.05) relative to CON (0.90 compared with 1.55). This data provide evidence to suggest that HFHC-induced obesity is associated with a decreased uterine response to OXT during labour. In contrast, accumulative dosing of PGF_2α_ during TNL and TL seemed to have no significant effect on the physiological parameters measured within animals sustained on the control and HFHC diet (Figures 6A–6D). It was not possible to fit a sigmoidal dose–response curve to increasing doses of PGF_2α_; however, during TNL, obese rats exposed to the HFHC diet were observed to have a blunted response to PGF_2α,_ but the response was similar between CON and HFHC-fed rats during labour (Figures 7A and 7B).

## DISCUSSION

Chronic feeding of a HFHC diet before and during pregnancy in the rat mirrored changes often associated with maternal obesity in women. These include a significant increase in body fat mass and circulating levels of total cholesterol [15–17]. Increasing body mass index can significantly depress human uterine contractility *ex vivo* [18] and cholesterol has been shown to play a key role in uterine contractile activity, as it causes significant utero-relaxant effects on both human and rat myometrium [19,20]. What was evident from the current study was that uterine tissue from control animals at both TNL and TL displayed regular biphasic plateau contractions with synchronized contraction and relaxation of uterine smooth muscle bundles [21]. In contrast, uterine tissue from HFHC-fed animals displayed a spike bundle contraction phenotype. Spike bundle contractions are multiphasic, resulting from asynchronous contraction and relaxation of uterine smooth muscle bundles [21]. This *ex vivo* contractility data provide evidence to suggest that the animals fed the HFHC diet are exhibiting poorly co-ordinated contractions, which agrees with previous reports that obese women have poor myometrial contractions during labour [18].

The aetiology of uterine dystocia is currently unknown and was the main reason for investigating the effects of adiposity on uterine expression of key CAP within the present study. Within myometrial cells are gap junction protein channels that allow cell–cell electro-coupling (synchronization) and calcium transfer [22]. CX-43 is a protein essential for gap junction formation and the significant decrease in CX-43 expression observed at TL in HFHC rats will decrease synchronization of the myocytes within the uterus. In parallel to CX-43 uterine expression of the phosphorylated protein pCX43 was also quantified. Phosphorylation of CX43 is negatively correlated with gap junction assembly and reduces cell-to-cell communication. Uterine expression of pCX43 decreased significantly with labour in lean control rats, but remained high during labour in HFHC rats. These differences in expression of CX43 and pCX43 could explain why HFHC rats exhibit asynchronous contractions. Cholesterol enrichment of cardiomyocyte culture medium leads to disassembly, phosphorylation and redistribution of CX-43 via protein kinase C [23]. Increased plasma cholesterol concentration exhibited by the HFHC animals may compromise uterine CX-43 expression in the current study through the same mechanism. Uterine expression of Cav-1, the cholesterol-rich structural protein of caveolae [8,24], decreased with the onset of labour in HFHC- and CON-fed rats. Caveolae are ω-shaped invaginations of the plasma membrane [8,24,25] that support a range of ion channels [19,20,26] and receptors [27–29] essential for regulating myometrial contractile activity. Interestingly, uterine expression of the OXTR differed between CON- and HFHC-fed rats. OXTR expression was significantly higher in HFHC-fed rats compared with CON. In human pregnancy, OXTR expression is lowest at early gestation, increases 12-fold by 37–41 weeks and then expressed maximally at labour onset [30]. The same is true in rats; OXTR expression is lower at mid gestation but increases significantly at parturition [31]. Uterine expression of CAP during pregnancy has been shown to be governed by sex steroids. Progesterone suppresses uterine CAP expression promoting uterine quiescence, whereas oestradiol up-regulates expression of the key contractile proteins and promotes contractile activity [22]. There is evidence to suggest that progesterone may promote uterine quiescence through synthesis of CX-43, but limit the trafficking of this protein to the plasma membrane and preventing formation of gap junctions essential to synchronous contractions [32]. In the current study, HFHC-induced adiposity led to a significant increase in plasma progesterone concentrations at term compared with CON rats and may offer an explanation of the occurrence of the asynchronous contraction phenotype and altered CAP expression within the uterus.

Overall, the present study illustrates the potential of an animal model of increased maternal adiposity to study the effects of obesity on parturition. To date the pathogenesis of uterine dystocia resulting from maternal obesity remains to be resolved. Potential mechanisms have been proposed and include (1) a shift in the progesterone and oestradiol ratio compromising uterine activation [3], (2) cholesterol altering the fluidity of the plasma membrane effecting integral protein expression [3,12] and possible utero-relaxant effects of adipokines and endocrine disruption resulting from increased white adipose tissue depots [3,33–35]. Using this model of maternal adiposity alongside biopsies of uterine tissue and plasma samples obtained from pregnant women of increasing body mass index can help unravel the mechanism(s) behind prolonged and dysfunctional labour associated with maternal obesity.

## CONCLUSION

The present study is the first to highlight the significant effects of an adiposity-inducing diet high in saturated fat and cholesterol on *ex vivo* uterine contractility and CAP expression during labour in rats. Chronic feeding of the HFHC diet led to aberrant changes in uterine contractile activity, uterine expression of CAPs and steroid production. These findings offer initial evidence of the aetiology of uterine dystocia that occurs in obese parturients. Further work is required to evaluate the effects of feeding a HFHC diet upon uterine contractility *in vivo* and to elucidate the mechanisms involved.

## Abbreviations

CAP: contractile-associated protein
Cav-1: caveolin-1
CON: control chow
COX-2: cyclooxygenase-2
CX-43: connexin 43
HFHC: high-fat, high-cholesterol
NHS: National Health Service
OXTR: oxytocin receptor
pCX43: phosphorylated form of connexin-43
PGF_2α_: prostaglandin F_2α_
TL: term labouring
TNL: term non-labouring
